# Near-infrared PAINT localization microscopy via chromophore replenishment of phytochrome-derived fluorescent tag

**DOI:** 10.1038/s42003-024-06169-7

**Published:** 2024-04-18

**Authors:** Kai Lu, Tetsuichi Wazawa, Tomoki Matsuda, Daria M. Shcherbakova, Vladislav V. Verkhusha, Takeharu Nagai

**Affiliations:** 1 SANKEN (The Institute of Scientific and Industrial Research), Osaka University, 8-1 Mihogaoka, Ibaraki, Osaka 567-0047 Japan; 2 Department of Genetics and Gruss-Lipper Biophotonics Center, Albert Einstein College of Medicine, Bronx, NY 10461 USA; 3 Medicum, Faculty of Medicine, University of Helsinki, Helsinki, 00290 Finland

**Keywords:** Fluorescence imaging, Biological fluorescence

## Abstract

Bacterial phytochromes are attractive molecular templates for engineering fluorescent proteins (FPs) because their near-infrared (NIR) emission significantly extends the spectral coverage of GFP-like FPs. Existing phytochrome-based FPs covalently bind heme-derived tetrapyrrole chromophores and exhibit constitutive fluorescence. Here we introduce Rep-miRFP, an NIR imaging probe derived from bacterial phytochrome, which interacts non-covalently and reversibly with biliverdin chromophore. In Rep-miRFP, the photobleached non-covalent adduct can be replenished with fresh biliverdin, restoring fluorescence. By exploiting this chromophore renewal capability, we demonstrate NIR PAINT nanoscopy in mammalian cells using Rep-miRFP.

## Abstract (web summary)

Reversible non-covalent interaction between a bacterial phytochrome-derived fluorescent protein and its biliverdin chromophore enables near-infrared localization nanoscopy in mammalian cells.

## Introduction

Super-resolution microscopy allows the observation of subcellular architectures beyond the diffraction limit of light. Among nanoscopy techniques, single-molecule localization microscopy (SMLM) offers high spatial resolution with simple optics and low hardware cost^1^. In SMLM, fluorophores are stochastically activated and sampled on single-molecule basis. Photoconvertible and photoswitchable fluorescent proteins (FPs) are the workhorses of SMLM tags, but typically require a total of 2–3 lasers for excitation, activation, and depletion of the fluorescent species^2,3^. The high spectral occupancy complicates the optics and limits multiplexing potential. We previously developed SPOON, a green FP that spontaneously blinks under single-laser illumination^4^. SPOON is simultaneously excited and switched off by 488 nm light at high irradiance. Off-state SPOON molecules rapidly and thermally relax back to the fluorescent state, therefore bypasses photoswitching and streamlines SMLM.

Alternatively, single-laser SMLM has been achieved by transient labeling using exchangeable probes^5,6^. A prominent example is Point Accumulation for Imaging in Nanoscale Topography (PAINT) and its variants, which rely on stochastic fluorophore binding to generate localizable single molecules^7–9^. Recently, FP-based PAINT was demonstrated using the bilirubin-activating protein UnaG^10^ and its brightness-enhanced mutant eUnaG^11^. UnaG is derived from Japanese eel muscle and fluoresces when non-covalently bound to the bilirubin chromophore^12^. UnaG is switched off by photobleaching of the bilirubin adduct and its release from the binding pocket, then switched on by rebinding to fresh bilirubin.

Here, we extend the spectrum of FP-based PAINT probes to the NIR by reporting a phytochrome-derived FP capable of chromophore renewal. This FP non-covalently binds the biliverdin IXα (BV). Under red light irradiation, the photobleached chromophore can be displaced by fresh BV. This reversible phytochrome-BV interaction leads to fluorescence turnover and single emitters compatible with the principle of PAINT localization microscopy.

## Results

### Phytochrome-derived near-infrared fluorescent protein with non-covalent biliverdin adduct

**Fig. 1:**
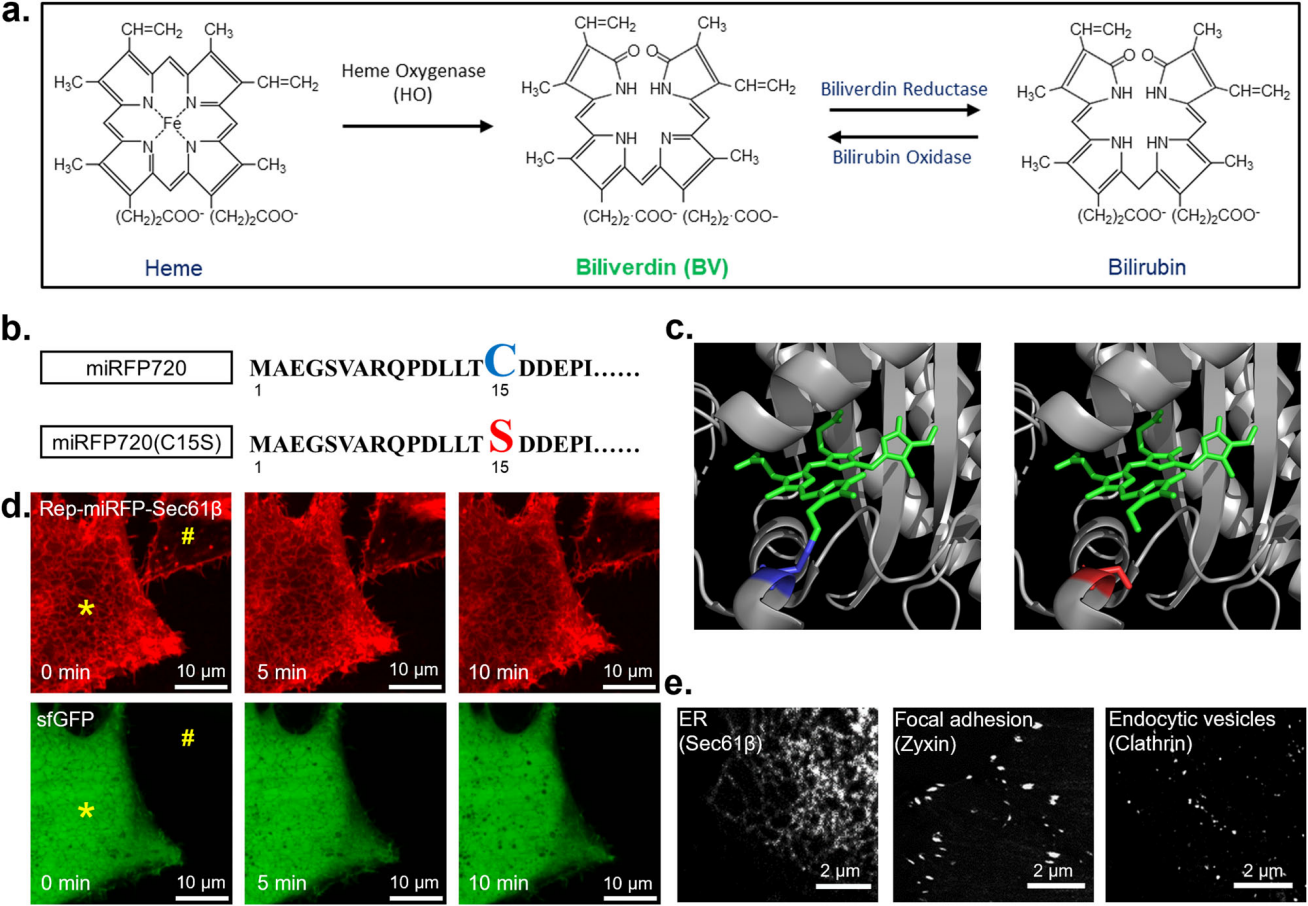
Fluorescence of miRFP720(C15S) that non-covalently interacts with biliverdin IXα. **a** Scheme of BV biosynthesis. Heme is converted to BV by heme oxygenase (HO) via cleavage of the heme ring at the α-methene bridge (orange rings in heme and BV structures). NADPH is the reducing agent in this reaction. Molecular oxygen enters the reaction and leads to the production of carbon monoxide (CO), and the iron is released from the BV molecule. BV is then converted to bilirubin by biliverdin reductase. In mammalian cells, BV and bilirubin coexist in an equilibrium maintained by bilirubin oxidase and biliverdin reductase. **b** N-terminal amino acid sequence alignment between miRFP720 and miRFP720(C15S). **c** Chromophore binding pocket of the bacterial phytochrome *Rp*BphP2 (PDB ID: 4R6L) that interacts covalently with BV (left panel). Disruption of the covalent interaction by C15S mutation (right panel schematic). Gray: RpBphP2. Green: BV. Blue: Cysteine 15. Red: Serine 15. **d** Live-cell confocal microscopy performed after incubation of HeLa cells with 30 µM BV for 24 h. Rep-miRFP was localized to the ER using Sec61β. Ubiquitous sfGFP was used as a transfection marker to identify positively transfected cells and expression level. Rep-miRFP-Sec61β and sfGFP were bicistronically expressed using a self-cleaving P2A peptide. Asterisk (*) indicates a positively transfected cell. Hash (#) indicates a non-transfected cell. **e** Fixed-cell confocal microscopy of miRFP720(C15S) localized to ER, focal adhesion sites, and endocytic vesicles with 0.5 µM BV in imaging buffer.

We developed the chromophore-renewable imaging tag by using the NIR FP miRFP720 as a molecular template^13^. miRFP720 was previously derived from *Rp*BphP2, a soluble bacterial phytochrome photoreceptor found in *Rhodopseudomonas palustris*^14^. Among phytochrome-derived FPs^15^, miRFP720 has the longest excitation (702 nm) and emission (720 nm) wavelengths. It covalently binds the BV chromophore via a single thioether bond at cysteine 15 residue, whereas other phytochrome-derived FPs, e.g., miRFP670, attach to BV by two thioether bonds. When expressed in mammalian cells, apo-miRFP720 took up endogenous BV produced through heme metabolism (Fig. 1a). The stable covalent bond between miRFP720 and BV resulted in constitutive NIR fluorescence when expressed in mammalian cells (Supplementary Fig. 1a). The fluorescence was not diminished by the efflux of unbound BV after permeabilization of the plasma membrane (Supplementary Fig. 1b).

It was previously reported that a mutant of the *Agrobacterium* phytochrome Agp1, which non-covalently interacts with BV, can recover its absorption spectrum from the photobleached state upon the addition of fresh BV^16^. Interestingly, photodamage primarily targeted the BV cofactor, leaving the phytochrome intact. Fresh BV displaced its photobleached counterpart from the binding pocket and restored a functional holoprotein. However, the fluorescence quantum yield of wildtype Agp1 is as low as 0.0002 (<1/300 of miRFP720)^17^, making it impractical as an bioimaging tag.

To develop a functional NIR FP that allows chromophore exchange, we disrupted the strong phytochrome-BV covalent bond by introducing the C15S mutation in miRFP720 (Fig. 1b, c). Using superfolder GFP (sfGFP) as a transfection marker (Supplementary Fig. 2a), we examined the fluorescence emission of miRFP720(C15S) in mammalian cells. In BV-free basal medium, the fluorescence of miRFP720(C15S) was barely observable in confocal microscopy (Supplementary Fig. 2b, c). This suggests that either the modified binding pocket failed to accommodate BV, or endogenous BV in cells was insufficient for the onset of ensemble fluorescence. We next increased the intracellular BV concentration by externally supplement 0.5 µM BV in imaging buffer after cell fixation. In addition, we localized miRFP720(C15S) to the endoplasmic reticulum (ER) using the Sec61β sequence (Supplementary Fig. 3a), to distinguish miRFP720(C15S) signal from BV-induced autofluorescence by localization pattern. ER-specific fluorescence was induced after adding BV (Supplementary Fig. 3b), indicating successful incorporation of BV by miRFP720(C15S). Using miRFP720(C15S) as a fusion tag, we acquired confocal microscopy images of ER, focal adhesions, and clathrin-coated vesicles in fixed cells supplemented with 0.5 µM BV (Fig. 1e). In live cells, the addition of 0.5 µM BV failed to immediately activate Rep-miRFP in minutes (Supplementary Fig. 3c), suggesting limited permeability of BV. Next, we investigated whether the chromophore could be delivered in live cells by long-term incubation with BV. To this end, untransfected HeLa cells were incubated for 24 h with up to 122 μM of BV in culture medium. Notably, BV solution can be weakly fluorescent and has an excitation peak at around 603 nm^18^. This allows the estimation of intracellular and extracellular BV levels by measuring fluorescence intensity using a sensitive EMCCD camera (Supplementary Fig. 4a). Intracellular BV increased monotonically with extracellular BV concentrations. Nevertheless, BV levels inside the cells were consistently lower than in the medium (Supplementary Fig. 4b). In addition, high fluorescence was observed on cell edges, which again confirmed that intact plasma membrane is a barrier for BV delivery (Supplementary Fig. 4a). We obtained time-lapse confocal image of ER-localized miRFP720(C15S) in live HeLa cells after incubation with 30 μM BV for 24 h (Fig. 1d), though non-specific fluorescence was induced by BV and observed on cell edges. We recently reported cell-level fluorescence of a non-covalent BV-binding serpin derived from *Boana punctata* (*Bp*BBS)^19^. To the best of our knowledge, miRFP720(C15S) is the first non-covalent BV-binding protein that functions as a fusion tag for the imaging of organelles and subcellular compartments.

### Reversible interaction between phytochrome and biliverdin enables fluorescence turnover

**Fig. 2:**
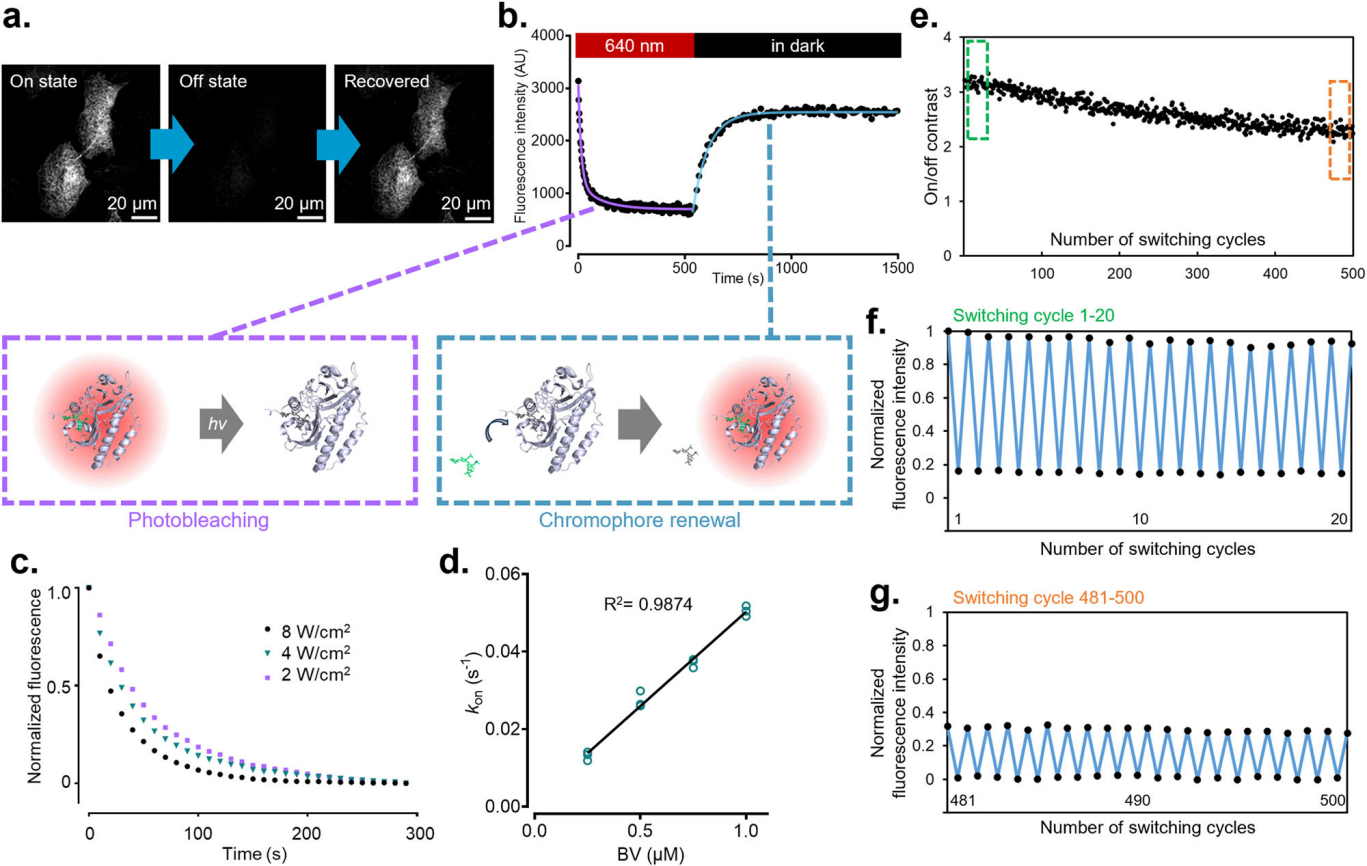
Fluorescence turnover of miRFP720(C15S) via chromophore renewal. **a** Confocal microscopic snapshots of miRFP720(C15S) localized to ER in an on-off-on switching cycle. **b** Switching kinetics of a complete on-off-on cycle of miRFP720(C15S). **c** On-to-off kinetics under continuous 640 nm laser irradiation at various power densities, measured in fixed HeLa cells supplemented with 0.5 µM BV. **d** Off-to-on fluorescence recovery speed of miRFP720(C15S) measured by *k*_on_ at various BV concentrations in fixed HeLa cells. Data points were fitted with a linear function Y = 0.04867·X + 0.0016. *n* = 3 independent experiments. **e** On/off contrast of miRFP720(C15S) during 500 switching cycles measured from time-lapse confocal images of fixed HeLa cells supplemented with 1 µM of BV. On/off contrast was calculated as the ratio of background-subtracted intracellular fluorescence intensity after and before recovery in the dark. Green box indicates cycle 1–20 in (**f**). Orange box indicates cycle 481–500 in (**g**). **f** Fluorescence of on- and off-state miRFP720(C15S) between switching cycle 1–20 in (**e**). **g** Fluorescence of on- and off-state miRFP720(C15S) between switching cycle 481–500 in (**e**). To display (**f**, **g**) in the same y-axis range, fluorescence in both panels was normalized between the highest and the lowest intensity in the entire time-lapse track shown in (**e**).

The β-barrel green FP UnaG incorporates its bilirubin chromophore through a non-covalent bond. It was recently found that the bilirubin adduct can detach from the UnaG protein backbone after photobleaching, freeing the binding cavity for de novo incorporate of fresh chromophore^10^. Bilirubin and BV are related bilins found in the heme metabolism pathway. To date, fluorescence turnover by the renewal of BV chromophore has not been reported. In fixed HeLa cells, the ensemble fluorescence of miRFP720(C15S) could be rapidly depleted by 640 nm laser irradiation (Fig. 2a). The decay curve was fitted with a bi-exponential function (magenta segment in Fig. 2b), suggesting the presence of two fluorescence states likely related to BV isomerization^20,21^. The on-to-off speed increased with the power density of 640 nm irradiation (Fig. 2c). Importantly, photobleached miRFP720(C15S) spontaneously recovered its fluorescence in the dark when fresh BV was available (Fig. 2a). The off-to-on curve was well fitted with a single-exponential function (blue segment in Fig. 2b). Increasing BV concentration linearly accelerated the fluorescence recovery process (Fig. 2d). The fluorescence turnover behavior of miRFP720(C15S) is similar to that of UnaG despite the drastic difference in their protein structures. It suggests that non-covalent protein-ligand interaction could be a general avenue that facilitates chromophore renewal.

Switchable FPs enable stochastic fluorescence activation which is the cornerstone of many super-resolution microscopy techniques. Compared to other switching mechanisms such as photoisomerization and protonation/deprotonation, chromophore renewal has the potential advantage of delaying the irreversible photodamage in time-lapse imaging. To this end, we switched miRFP720(C15S) for 500 cycles in fixed HeLa cells supplemented with 1 μM BV, then measured the on/off contrast during the 10-hour duration (Fig. 2e). Notably, the ensemble fluorescence remained detectable at the end of the experiment (Fig. 2f, g). We named this chromophore replenishable miRFP720 mutant as Rep-miRFP, because of the fluorescence turnover property.

### Near-infrared PAINT localization microscopy based on the renewal of biliverdin chromophore

**Fig. 3:**
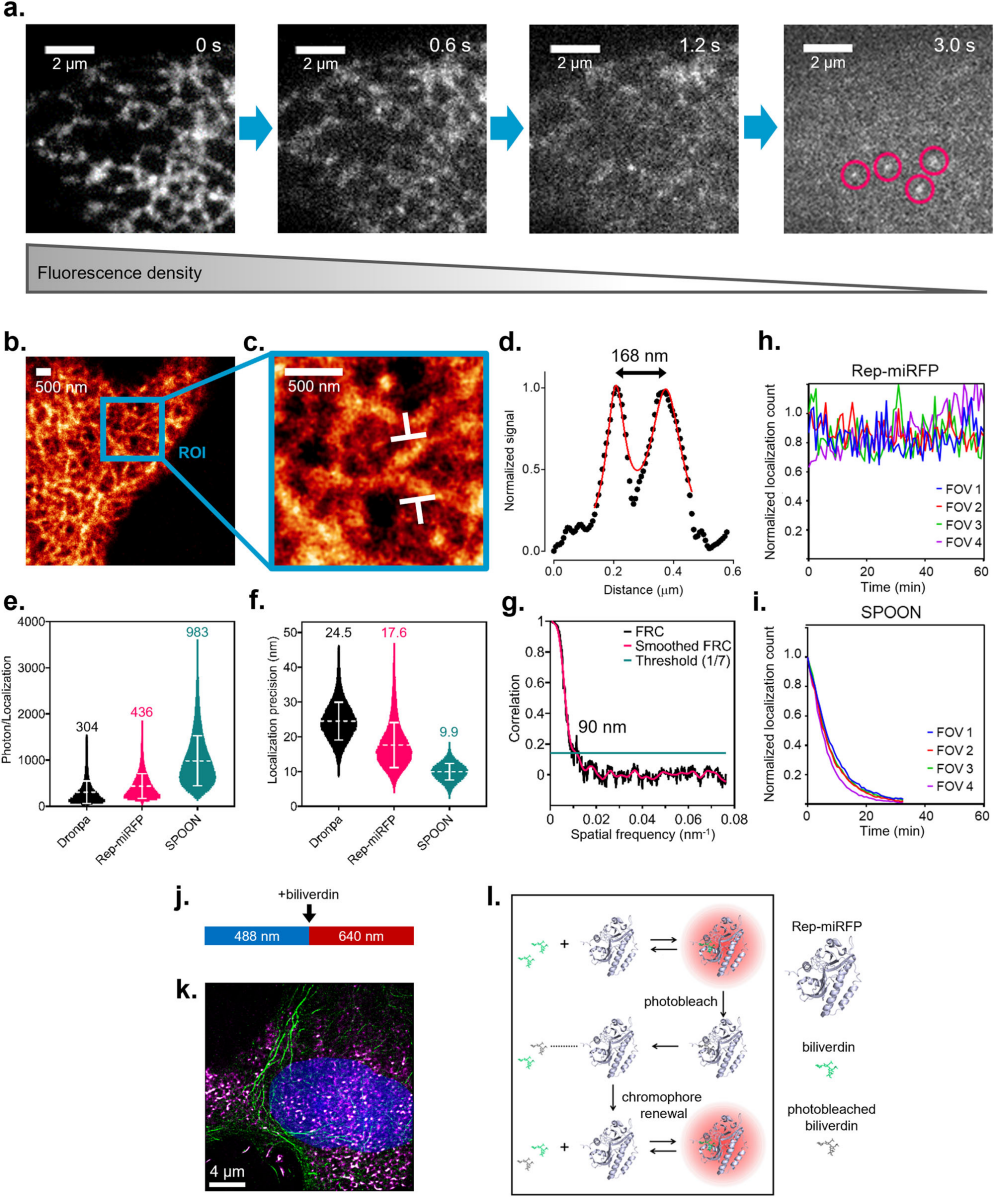
PAINT localization microscopy in mammalian cells using Rep-miRFP. **a** Reduction of Rep-miRFP signal from ensemble fluorescence to single emitters under continuous 640 nm irradiation in the presence of 0.5 µM BV. Time-lapse images were acquired in fixed COS-7 cells expressing Rep-miRFP-Sec61β (localized to ER) and supplemented with 0.5 µM BV. **b** Reconstructed SMLM image of ER labeled with Rep-miRFP-Sec61β. Pixel size, 6.5 nm. **c** Zoom-in on the region of interest (ROI) in (**b**). **d** Intensity line profile measured between the white arrows in (**c**). The double peak was fitted with the sum of two Lorentzian functions. **e** Number of photons per localization. Data were presented as a scatter plot of localized molecules, with mean ± SD. Mean photon values were shown above each scatter plot. **f** Localization precision. Data were presented as a scatter plot of localized molecules, with mean ± SD. Mean localization precision values were shown above each scatter plot. **g** Fourier ring correlation (FRC) for evaluating the lateral resolution of SMLM image obtained with Rep-miRFP. The single-emitter track used for reconstructing (**b**) was split into two stacks containing odd or even number of frames, and then reconstructed into two statistically independent SMLM images for FRC analysis. A FRC resolution of 90 nm was estimated by the inverse of the spatial frequency, at the intercept of the FRC curve and the threshold of 1/7 correlation. **h** Time trajectories of Rep-miRFP localization count during 60 min of SMLM image acquisition. **i** Time trajectories of SPOON localization count during 30 min of SMLM image acquisition. For (**h**, **i**), each data point represents the total number of emitters localized in a 50-s window. Data were normalized between the highest count and zero. Data from 4 different fields of view (FOV) were plotted for Rep-miRFP and SPOON, respectively. **j** Image acquisition schedule of two-color SMLM using Rep-miRFP (NIR channel) and SPOON (green channel). **k** Multiplexed SMLM image of ER and F-actin using Rep-miRFP and SPOON. The nucleus was counterstained with Hoechst and imaged by diffraction-limited microscopy. **l** Schematic of chromophore renewal in Rep-miRFP. Fresh BV displaces its photobleached counterpart from the binding cavity and restores a fluorescent holo-phytochrome.

We next examined whether the chromophore renewal of Rep-miRFP could be exploited for PAINT localization microscopy to super-resolve a subcellular structure. We applied highly inclined and laminated optical sheet (HILO) illumination using a 640 nm laser, and imaged fixed COS-7 cells expressing Rep-miRFP localized to the ER. COS-7 was chosen because its flat morphology well complements HILO illumination for capturing the ER structure. In imaging buffer supplied with 0.5 µM BV and under 640 nm laser illumination at 28.9 W cm^−2^, the ensemble fluorescence of Rep-miRFP was rapidly reduced to single emitters in seconds (Fig. 3a and Supplementary Movie 1). Single emitters were sustained in an equilibrium between photodepletion and de novo formation of Rep-miRFP holoprotein. Interestingly, we noticed a background fluorescence prominent in the intracellular compartment, which was not observed in the cell-free regions (Supplementary Fig. 5). This background was also detected in untransfected live cells after plasma membrane permeabilization and the influx of BV (Supplementary Movie 2), indicating that BV was the source of this background fluorescence. Upon a closer examination of confocal images under high magnification, we also found that the non-specific fluorescence preferentially highlighted the mitochondria (blue arrow in Supplementary Fig. 6a). These results are consistent with reports of BV accumulation at mitochondria^22^ and binding with proteins partners such as albumin^23^. The non-specific fluorescence negatively impacts the fluorescence contrast of Rep-miRFP. In the absence of BV-induced background, the on/off contrast of Rep-miRFP was twice as high as that of Dronpa (Supplementary Fig. 7), a well-documented photoswitchable green FP commonly used for optical highlighting and super-resolution microscopy^24^. In the presence of BV-induced background, the Rep-miRFP signal was obscured and the on/off contrast was compromised (Supplementary Fig. 7).

The BV-induced background has relatively little impact on confocal microscopy, because the ensemble fluorescence of the holoprotein is usually dominant. In SMLM, fixed pattern background could reduce the accuracy of single-molecule localization and the fidelity of super-resolution images^25^. We sought to eliminate the background in single-molecule image data prior to emitter localization. The background cannot be removed by an emission filter due to spectral overlap with Rep-miRFP. However, we observed in time-lapse images that while Rep-miRFP was rapidly switched off by the 640 nm laser, fluorescence intensity of the fix pattern background was stable on the time scale of typical image acquisition and inert to the irradiation (Supplementary Fig. 6b). This difference in temporal behavior allowed the extraction of single-emitter foreground, by using advanced background subtraction algorithms such as SMALL-LABS^26^ and WindSTORM^27^. Although their detailed implementation differs, both SMALL-LABS and WindSTORM algorithms are designed to separate fast-changing foreground from slow-changing background. We processed the single-molecule image data of Rep-miRFP using SMALL-LABS in MATLAB. Compared to the raw images, the single emitters in the output were no longer obscured by the fix pattern background (Supplementary Fig. 5 and Supplementary Movie 3). We reconstructed super-resolution image of the ER using the background-subtracted data (Fig. 3b). Line profile analysis revealed a 168 nm peak-to-peak distance between separated structures (Fig. 3c, d), which surpassed the diffraction limit. The lateral resolution was also estimated by Fourier ring correlation (FRC)^28,29^, which showed a FRC resolution of 90 nm (Fig. 3g). Next, we compared the single-emitter performance of Rep-miRFP with β-barrel FPs. Each localized Rep-miRFP molecule emitted an average number of 436 photons (Fig. 3e). In comparison, the photon budget of Dronpa, an early generation photoswitchable FP, was estimated to be 304 photon/localization. On the other hand, the very bright SPOON emitted 983 photon/localization. The photon budget of SPOON is comparable to other state-of-the-art SMLM tags such as mEos3.2 and mMaple^2,3^, which led to a high localization precision of 9.9 nm. The localization precision of Rep-miRFP was 17.6 nm (Fig. 3f), worse than that of SPOON but superior to Dronpa (24.5 nm). Notably, due to chromophore renewal, the number of detectable Rep-miRFP single emitters remained largely unchanged after 60 min of continuous 640 nm laser irradiation (Fig. 3h). In comparison, the single emitters of SPOON were almost completely exhausted after 30 min, due to photobleaching by the 488 nm laser (Fig. 3i). In contrast to fixed cells that have unblocked access to free BV, live-cell SMLM imaging was limited by the inability of medium BV to rapidly diffuse across the plasma membrane. This was demonstrated by the quick exhaustion of diffusive intracellular BV and localizable molecules, even after incubating live cells with 30 µM BV for 24 h (Supplementary Fig. 8).

Rep-miRFP emits in the NIR spectrum which is not occupied by existing photoconvertible and photoswitchable FPs, making it flexible for multiplexed super-resolution imaging. We performed two-color SMLM using the combination of Rep-miRFP and SPOON. Rep-miRFP-Sec61β and Lifeact-SPOON were co-expressed in COS-7 cells by co-transfection. Images of the two fluorescence channels were acquired sequentially in fixed cells. The SPOON channel was acquired first under 488 nm illumination. After adding BV at a final concentration of 0.5 μM, the Rep-miRFP channel was acquired next under 640 nm illumination (Fig. 3j). Axial chromatic aberration was measured and compensated (Supplementary Fig. 9). As a result, multiplexed SMLM of the ER and F-actin was obtained (Fig. 3k).

## Discussion

Proteins using BV as a fluorescent cofactor have become attractive templates for the development of FPs, because it is challenging to cover the NIR spectrum by using β-barrel FPs. Extensive mutagenesis has led to some of the most redshifted β-barrel FPs including mKelly1 (maximum λ_em_ = 656 nm)^30^ and mGarnet2 (maximum λ_em_ = 671 nm)^31^. In comparison, wildtype BV-binding proteins naturally have longer emission wavelength, e.g., Sandercyanin (maximum λ_em_ = 675 nm)^32^ and *Bp*BBS (maximum λ_em_ = 691 nm)^19^. Here, we have described a phytochrome-derived FP that non-covalently and reversibly interacts with BV (Fig. 3l). Previously, UnaG was reported for PAINT localization microscopy in green channel. Rep-miRFP expands the color palette of FP-based PAINT probes. Our finding suggests that chromophore renewal might be a generalizable approach for developing genetically encoded PAINT probes from a larger variety of templates in the future, including BV-binding phytochromes^13,15,33^, cyanobacteriochromes^34,35^, phycobiliproteins^36^, lipocalin^32^ and serpins^19^.

To the best of our knowledge, Rep-miRFP is the first non-covalent BV-binding protein that functions as a fusion tag for fluorescence microscopy. Two other promising candidates in this class are: Sandercyanin, a tetrameric lipocalin found in walleye fish^32^; *Bp*BBS, a serpin found in polka-dot tree frog^19^. Although the fluorescence of these two were documented, their functionality as bioimaging tags has not been well explored.

Currently, Rep-miRFP has two major shortcomings. First, the negatively charged carboxylates of BV limits its membrane permeability^37^ and the utility of Rep-miRFP for live-cell imaging. BV variants with higher membrane permeability have been synthesized by modifying the carboxylates to create more neutrally charged molecules. The resulting biliverdin dimethyl ester (BVMe_2_)^36^ and BV-PEG-FAM^37^ can be incorporated by the phycobiliprotein-derived FP smURFP in live cells and tissues. However, bacterial phytochromes use the carboxylic acids of BV as a recognition motif, thus cannot incorporate BVMe_2_. It remains to be seen whether chromophore-renewable FPs could be derived from templates that naturally tolerate BVMe_2_, and whether molecular evaluation of phytochromes could lead to a modified chromophore binding pocket that accommodates BVMe_2_.

Second, Rep-miRFP is less bright than the best-in-class photoswitchable FPs in SMLM. Typically, the brightness of BV-binding proteins is improved by reducing chromophore flexibility and increasing the overall protein rigidity to suppress nonradiative transition^38^. By extensive mutagenesis and screening, the fluorescence quantum yield of a monomeric cyanobacteriochrome, miRFP670nano3, recently reached a record high of 0.185^35^. Stabilizing the BV binding pocket while preserving the chromophore exchange ability of Rep-miRFP could be a challenging but exciting avenue to go down in the future.

## Methods

### Fluorescent proteins

The gene encoding miRFP720 was cloned into the pcDNA3.0 vector (Invitrogen/Thermo-Fisher Scientific) between the BamHI and EcoRI restriction sites. The C15S point mutation was introduced in miRFP720 by inverse PCR using the KOD FX Neo DNA polymerase (Toyobo Life Sciences). The PCR product was digested by DpnI at 37 °C overnight to remove the template DNA, followed by T4 ligation (Promega). SPOON was previously developed in our lab by mutagenesis of Dreiklang ^39^. Dronpa was a gift from Atsushi Miyawaki at RIKEN Center for Brain Science.

### Construction of mammalian expression plasmids

For the fluorescent labeling of organelles and subcellular compartments, ER localization was achieved by fusing Sec61β to the C-terminus of FPs and insertion into pcDNA3.0 between BamHI and EcoRI restriction sites using In-Fusion cloning (Takara Bio). Sec61β fragment was obtained from pAc-GFPC1-Sec61β (Addgene #15108). For labeling focal adhesion sites, emiRFP703 between AgeI and NotI restriction sites in pzyxin-emiRFP703 (Addgene #136568) was swapped with Rep-miRFP. For labeling clathrin-coated vesicles, Kohinoor between NheI and BglII restriction sites in Kohinoor-Clathrin (Addgene #67773) was swapped with Rep-miRFP. For labeling the nucleus, Phamret between BamHI and EcoRI restriction sites in Phamret-H2B (Addgene #51955) was swapped with SPOON or miRFP720. For bicistronic expression of sfGFP and NIR FPs, the two domains were linked by a self-cleaving P2A peptide and inserted into pcDNA3.0 between BamHI and EcoRI restriction sites.

### Cell culture and transfection

HeLa and COS-7 cells were cultured in low-glucose Dulbecco’s modified Eagle medium (DMEM; Sigma-Aldrich) supplemented with 10% fetal bovine serum (FBS; Biowest) at 37 °C in a 5% CO_2_ incubator. Cells were seeded on home-made 35 mm glass-bottom dishes and grown to 50–60% confluence. For transfection, 2 μg of plasmid (or 1 μg of each plasmid for co-expression) was mixed with 5.0 μg of polyethylenimine MAX (Polysciences) in 200 μL of Opti-MEM medium (Thermo Fisher Scientific). The mixture was incubated for 20 min at room temperature and then added dropwise to the cell culture. The medium was refreshed after 6 h, and the cells were cultured for 24–48 h before observation.

### Confocal microscopy

For live-cell confocal microscopy, cells were immersed in DMEM/F-12 without phenol red (Thermo Fisher Scientific). For fixed-cell confocal microscopy, the cells were treated with 4% paraformaldehyde for 20 min at room temperature, then washed three times with phosphate buffered saline. Cells were imaged with a Dragonfly 200 spinning disk confocal microscope (Andor) equipped with an iXon Ultra EMCCD camera (Andor), quad-band 405/488/561/640 dichroic mirror, Plan Apo Lambda 60×/1.40 oil immersion or Plan Apo Lambda S 100×/1.35 silicone oil immersion objective lens (Nikon). The Dragonfly 200 spinning disk confocal microscope and iXon Ultra EMCCD camera were controlled by Fusion 2.3.0.45 (Andor). NIR FPs were excited with 640 nm laser and the fluorescence collected through a 725/40 emission filter (Semrock). Switching kinetics of Rep-miRFP was measured in time-lapse using the same setup. Green FPs including sfGFP and Dronpa were excited with 488 nm laser and their fluorescence was collected through a 521/33 emission filter (Semrock).

### PAINT localization microscopy

COS-7 cells were transfected with sfGFP-P2A-Rep-miRFP-Sec61β/pcDNA3.0. sfGFP was used as a transfection marker to identify transfected cells and expression level. The single-molecule imaging system was based on an inverted microscope (ECLIPSE Ti, Nikon) equipped with an sCMOS camera (ORCA-Flash4.0, Hamamatsu Photonics), a quad-band 405/488/561/640 dichromic mirror and a 732/68 and 525/45 emission filter (Semrock). The ORCA-Flash4.0 sCMOS camera was controlled by HCImageLive 4.3.1.33 (Hamamatsu Photonics). Objective-based HILO illumination was achieved using a 640 nm laser (OBIS 640 nm LX 100 mW, Coherent) and an Apo TIRF 100×/1.49 oil-immersion objective lens (Nikon). Single-molecule image track was acquired with an exposure time of 200 ms at a frame rate of 5 Hz. The irradiance of 640 nm laser at the sample plane was 28.9 W cm^−2^. Laser power was measured with a laser power meter (power meter console, PM400, Thorlabs; photodiode sensor, S120VC, Thorlabs). Size of the illumination area was measured with an objective micrometer (OB-M#, 1/100, Olympus).

### Reconstruction of super-resolution images

The raw single-molecule image track of Rep-miRFP was processed by the SMALL-LAB algorithm^26^ in MATLAB 9.8 to remove the heterogeneous background found inside fixed cells after the addition of BV. The workflow of background subtraction followed the user guide (https://github.com/BiteenMatlab/SMALL-LABS). First, an initial round of crude background subtraction was performed before emitter detection. In each frame, the low-frequency background that changed slowly over time was globally removed by calculating and subtracting a temporal mean image over a sliding window of 300 frames (1 min). Second, approximated detection of emitters was performed after the last step. Third, for each detected emitter, its unique local background was calculated by temporal searching for neighboring frames in which this emitter was in off state and not obscured by other on-state molecules in the diffraction-limited region. Fourth, the true local background specific to each detected molecule was subtracted. Fifth, the background-subtracted single molecules were localized with diffraction-unlimited precision by gaussian fitting. Lastly, SMLM images were reconstructed from 10,000 frames using the coordinates of localized molecules obtained in the last step.

### Image analyses and Fourier ring correlation

Image data were analyzed using ImageJ 1.54 f and Fiji software (https://fiji.sc). Fourier ring correlation (FRC) analysis was performed with an ImageJ plugin (https://github.com/BIOP/ijp-frc). The single-molecule track was split into two stacks containing odd or even number of frames, reconstructed into two statistically independent SMLM images, and then fed into the plugin for FRC analysis. The spatial resolution was estimated by the inverse of the spatial frequency at the intercept of the FRC curve and the threshold of 1/7 correlation.

### Multiplexed SMLM

COS-7 cells were co-transfected with Rep-miRFP-Sec61β/pcDNA3.0 and Lifeact-SPOON/pcDNA3.0, then fixed. The same microscope setup for single-color SMLM was used, except for an additional light path for 488 nm laser. Sequential image acquisition was performed in the order of green channel followed by NIR channel. Objective-based HILO illumination of SPOON was achieved using a 488 nm laser (OBIS 488 nm LS 150 mW, Coherent). First, the green channel single-molecule image track was acquired without adding BV, with an exposure time of 200 ms at a frame rate of 5 Hz using a 525/45 emission filter (Semrock). The power density of 488 nm laser at the sample plane was 54.5 W cm^−2^. Subsequently, BV was added at a final concentration of 0.5 µM and the NIR channel single-molecule image track was acquired under 640 nm laser irradiation. SMLM image of SPOON was reconstructed from 10,000 frames using ThunderSTORM v1.3^40^, using ‘difference of Gaussians’ filter for particle detection, ‘integrated Gaussian’ for PSF model and ‘maximum likelihood estimation’. Protein PAINT image of Rep-miRFP was reconstructed as described in the previous section.

### Statistics and reproducibility

Curve fitting was performed using GraphPad Prism 9.3.1. Data obtained from each experiment were expressed as the mean ± SD. The sample sizes were described in each figure legend.

### Reporting summary

Further information on research design is available in the Nature Portfolio Reporting Summary linked to this article.

### Supplementary information

Supplementary Information

Description of Additional Supplementary Files

Supplementary Data

Supplementary Movie 1

Supplementary Movie 2

Supplementary Movie 3

Reporting Summary

## Data Availability

The source data for the main graphs are provided in Supplementary Data. All other supplementary data in this study are available from the corresponding author on reasonable request.
