# Supplementary material for: Near-infrared PAINT localization microscopy via chromophore replenishment of phytochrome-derived fluorescent tag
Source: Commun Biol. 2024 Apr 18;7:473. doi: 10.1038/s42003-024-06169-7 (PMC11026395; doi:10.1038/s42003-024-06169-7)
Supplement: Supplementary file 1 — Supplementary Information [file 42003_2024_6169_MOESM1_ESM.pdf]

Supplemental Information

Near-infrared PAINT localization microscopy via chromophore replenishment of phytochrome-derived fluorescent tag

Kai Lu[1], Tetsuichi Wazawa[1], Tomoki Matsuda[1], Daria M. Shcherbakova[2], Vladislav V. Verkhusha[2,3] and Takeharu Nagai[1,*]

[1]*SANKEN (The Institute of Scientific and Industrial Research), Osaka University, 8-1 Mihogaoka, Ibaraki, Osaka 567-0047, Japan*

[2]*Department of Genetics and Gruss-Lipper Biophotonics Center, Albert Einstein College of Medicine, Bronx, NY 10461, USA*

[3]*Medicum, Faculty of Medicine, University of Helsinki, Helsinki 00290, Finland*

*Correspondence should be addressed to ng1@sanken.osaka-u.ac.jp (T.N.)

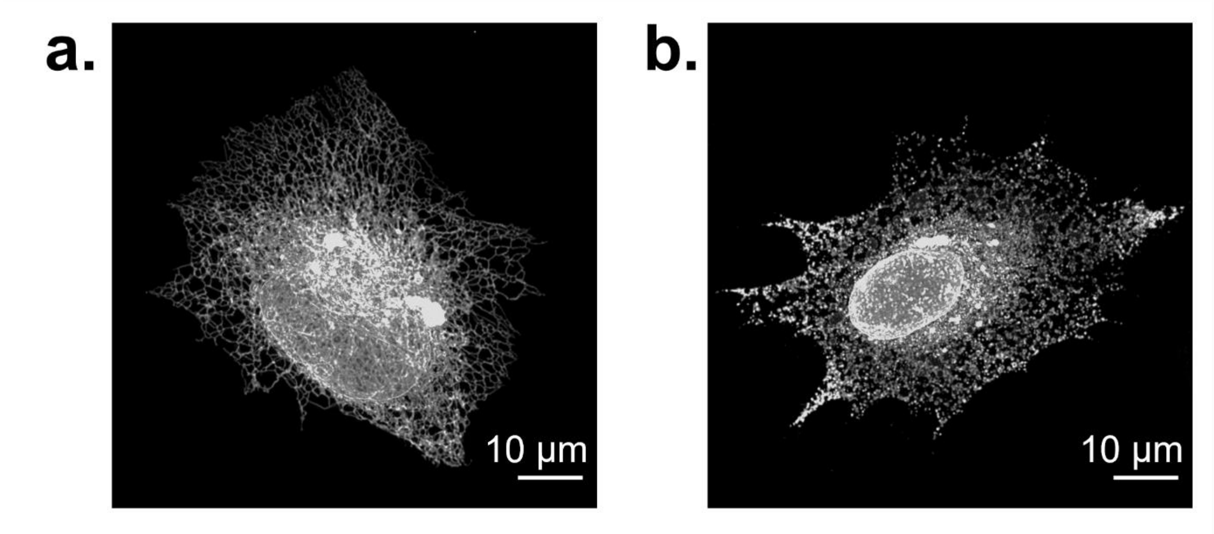

Supplementary Figure 1. Fluorescence images of miRFP720 that covalently binds BV. **a.** Confocal image of a live COS-7 cell expressing miRFP720-Sec61β. **b.** Confocal image of miRFP720-Sec61β in a live COS-7 cell after permeabilization of the plasma membrane by digitonin. Note that the originally intact ER structure was disrupted after the permeabilization and cytosol leakage.

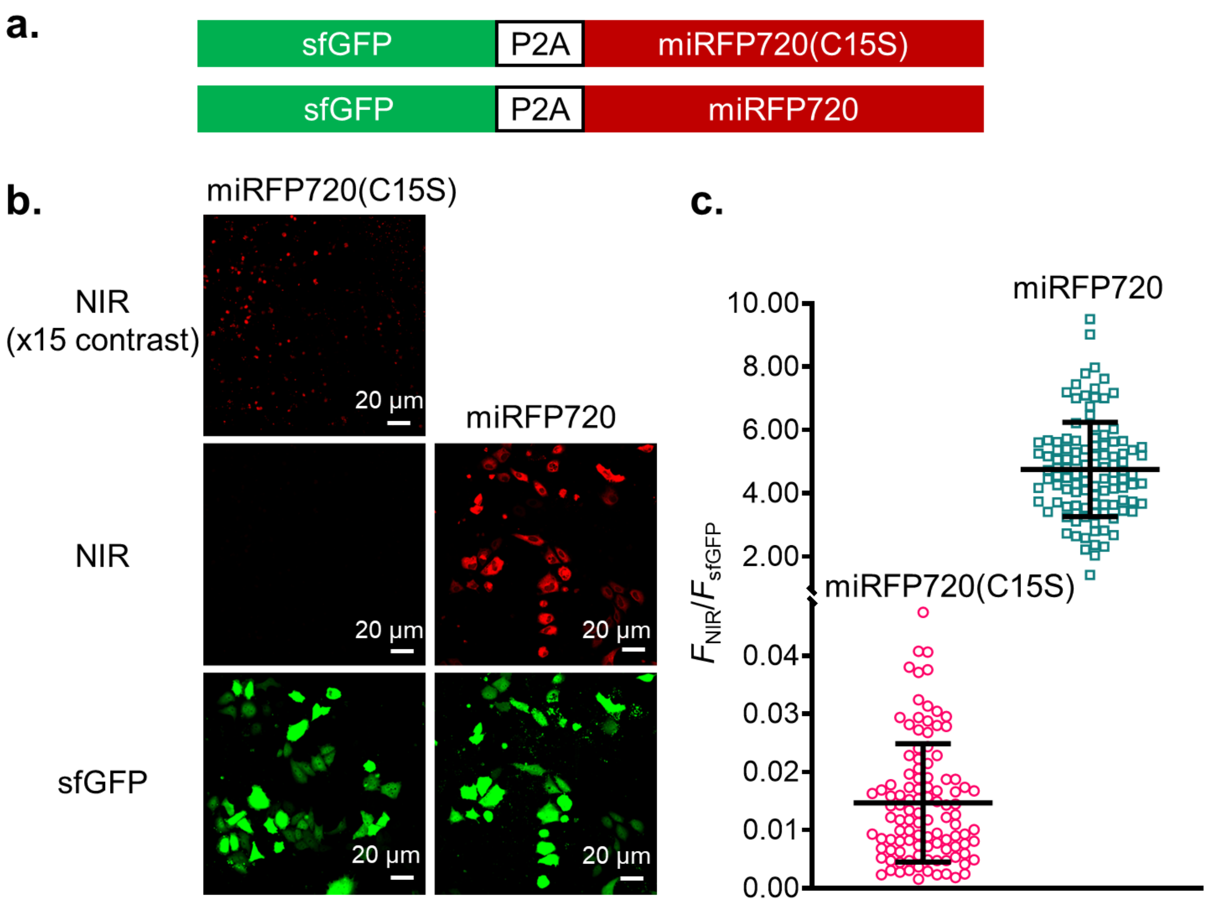

Supplementary Figure 2. Ensemble fluorescence of miRFP720(C15S) compared to miRFP720 in live mammalian cells. **a.** Bicistronic expression of miRFP720(C15S)/miRFP720 together with sfGFP by using a self-cleaving peptide P2A. **b.** Confocal microscopy of miRFP720(C15S) and miRFP720 in live HeLa cells. Imaging was performed in phenol-free DMEM/F12 basal medium without the addition of BV. sfGFP was used as a transfection marker to identify transfected cells and access expression level. **c.** Fluorescence intensity of miRFP720(C15S) and miRFP720 in live HeLa cells without the addition of BV. NIR fluorescence intensity was normalized by sfGFP to compensate the difference in expression level. n = 107 cells for miRFP720(C15S). n = 111 cells for miRFP720.

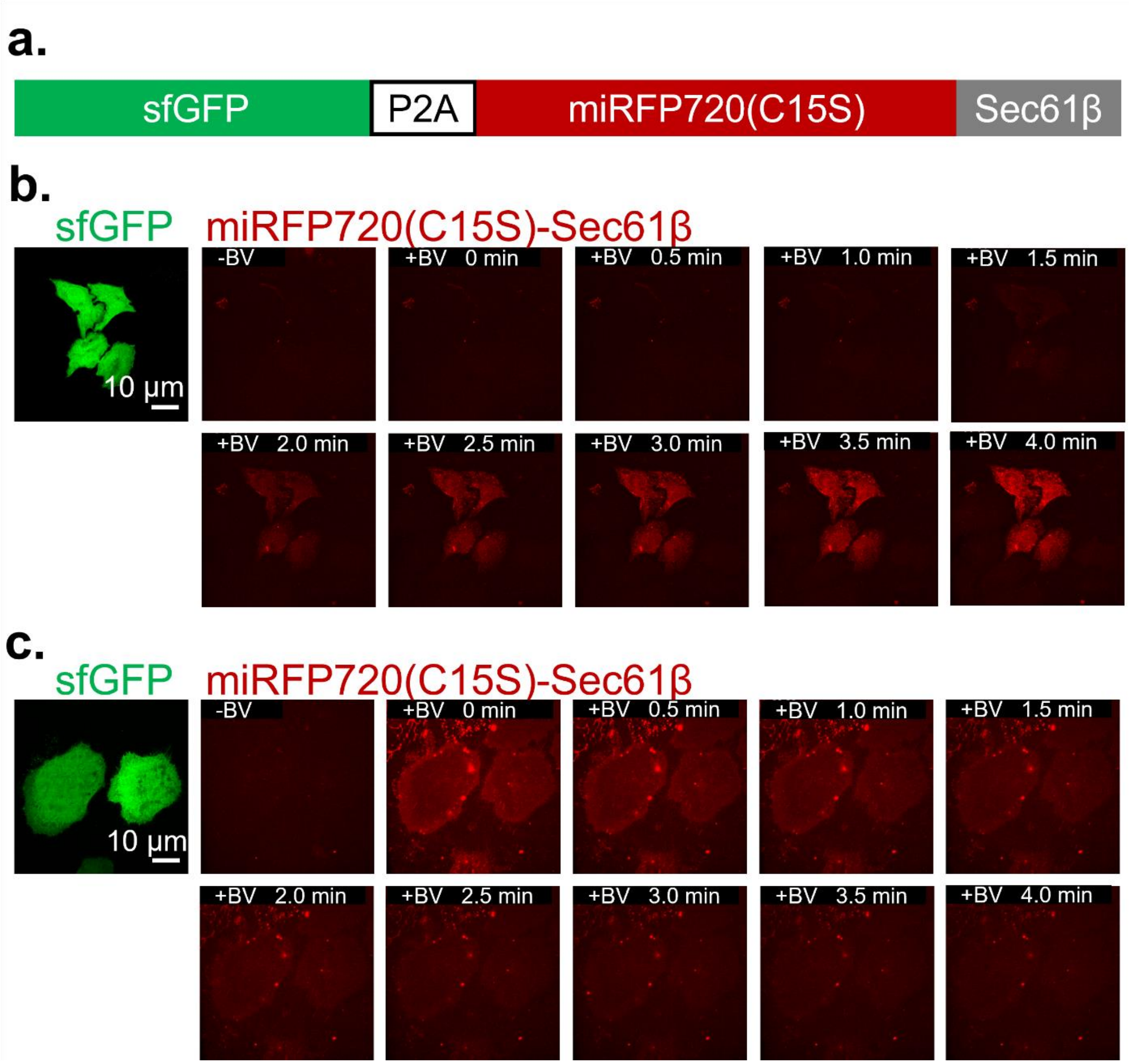

Supplementary Figure 3. Induction of miRFP720(C15S) fluorescence in fixed mammalian cells by the addition of BV. **a.** Bicistronic expression of miRFP720(C15S)-Sec61β together with sfGFP by using a self-cleaving peptide P2A. **b.** ER-specific fluorescence was induced in fixed HeLa cells after the addition of 0.5 μM BV. **c.** No ER-specific fluorescence was observed in live HeLa cells with intact plasma membrane after the addition of 0.5 μM BV. Note that a brief flash of nonspecific fluorescence was observed at the cell edges immediately after BV addition, indicating the intact plasma membrane as a barrier to BV delivery.

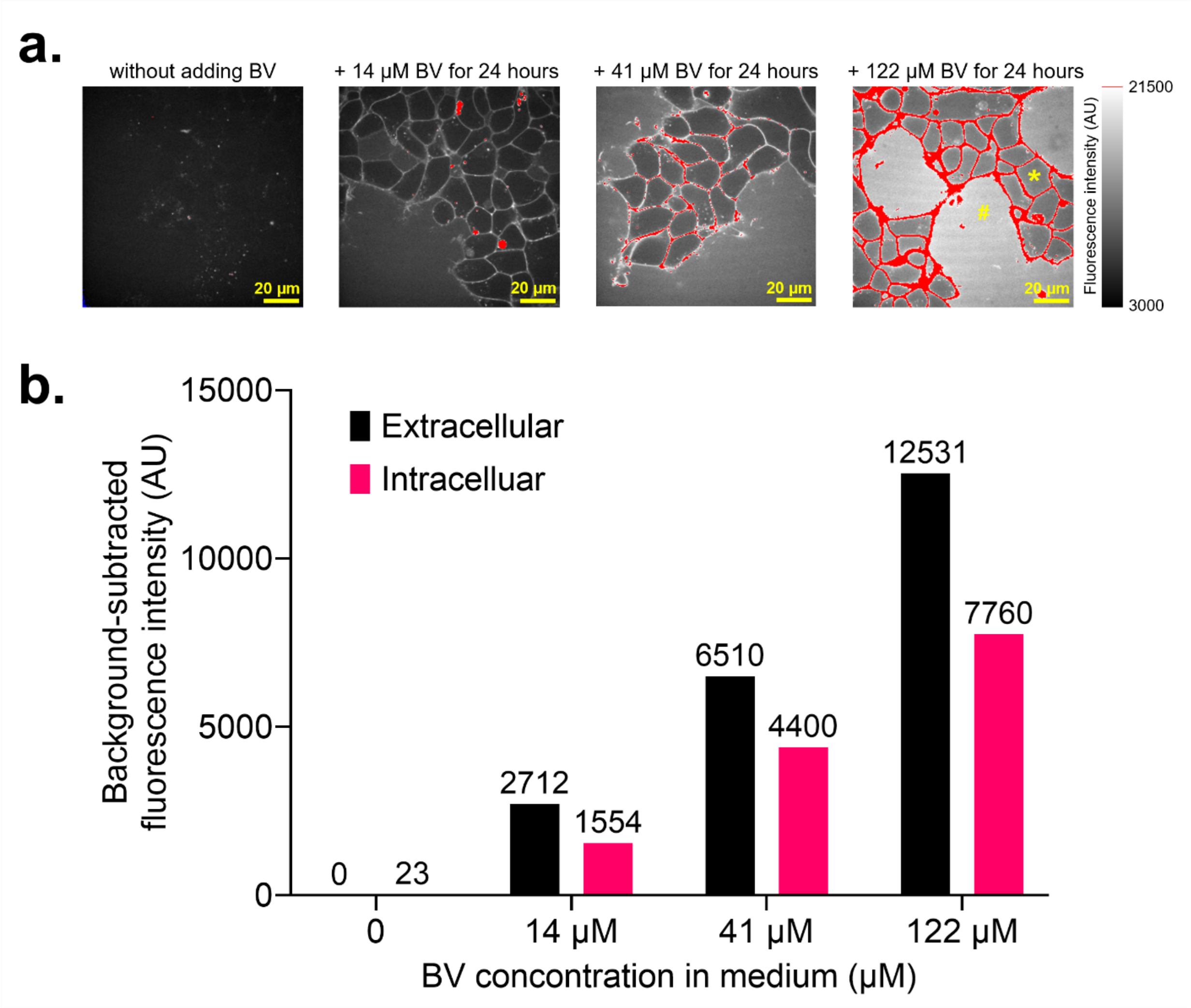

Supplementary Figure 4. Chromophore delivery by long-term incubation of live mammalian cells with BV. **a.** Confocal microscopy of untransfected live HeLa cells incubated for 24 hours with various concentrations of BV in the culture medium. Asterisk (*) indicates an intracellular region, hash (#) indicates an extracellular region. Samples were excited with a 640 nm laser. Fluorescence was collected with a 700/75 emission filter and an EMCCD camera. **b.** Quantification of intracellular and extracellular fluorescence intensity in panel a. Background was measured as extracellular fluorescence intensity in the medium when no BV was added, which was subtracted from the data. Intensity values are shown above each bar.

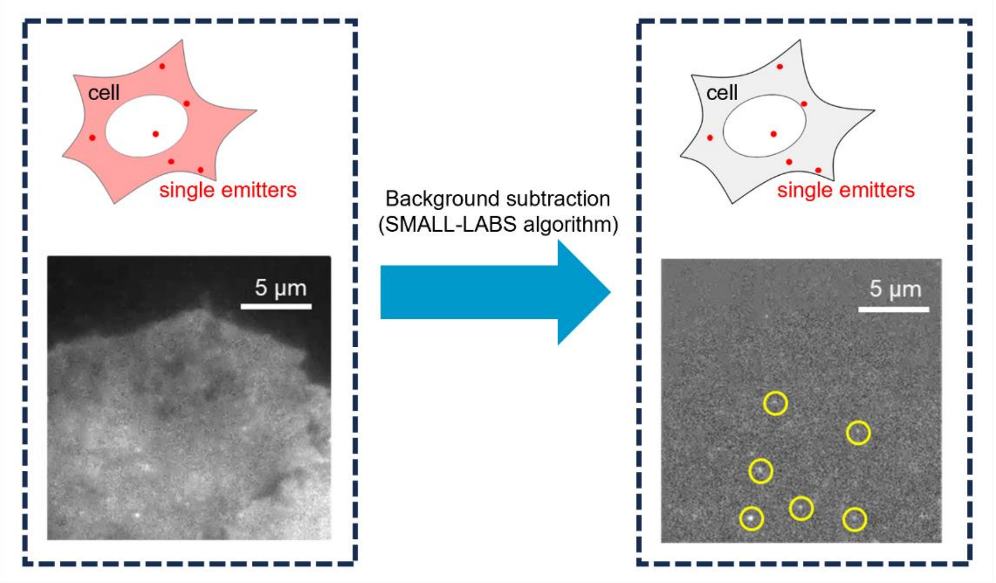

Supplementary Figure 5. Subtraction of non-specific intracellular fluorescent background in SMLM image data. Upper row: Schematics of single emitters (red dots) in a cell before and after background subtraction. Lower row: a single image frame containing single emitters before and after background subtraction with SMALL-LAB algorithm.

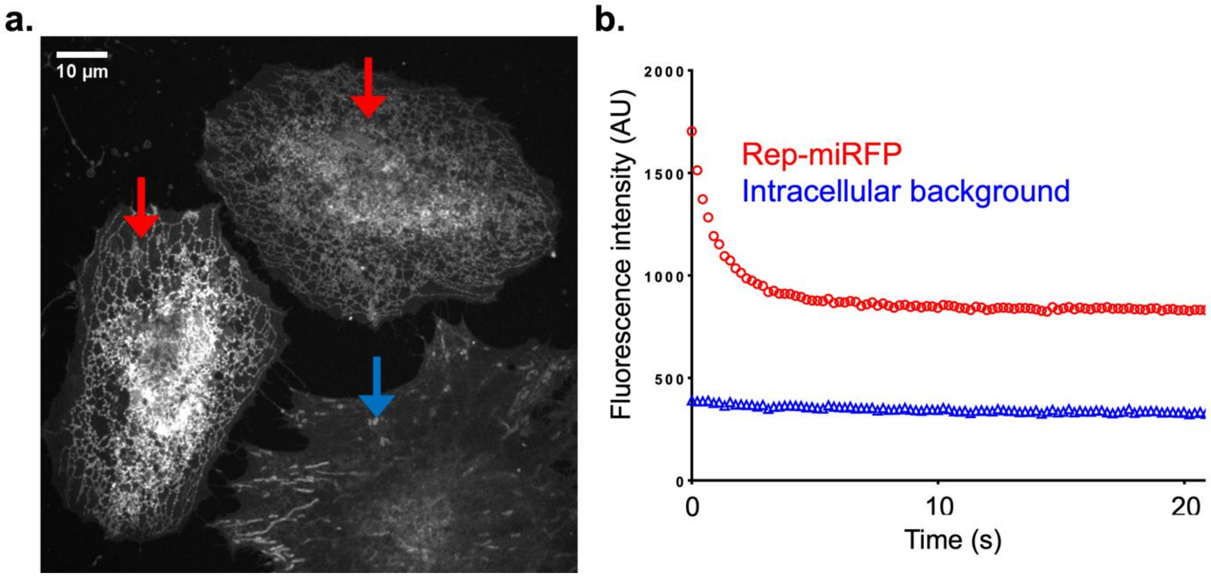

Supplementary Figure 6. Specific Rep-miRFP fluorescence and non-specific intracellular fluorescence induced by BV. **a.** High magnification confocal image of fixed COS-7 cells supplemented with 0.5 µM BV (emission filter 700/75). Red arrows: Cells expressing ER-localized Rep-miRFP-Sec61β. Blue arrow: Non-transfected cell. **b.** Time trajectories of ER-specific Rep-miRFP fluorescence and intracellular background under continuous 640 nm laser irradiation.

a.

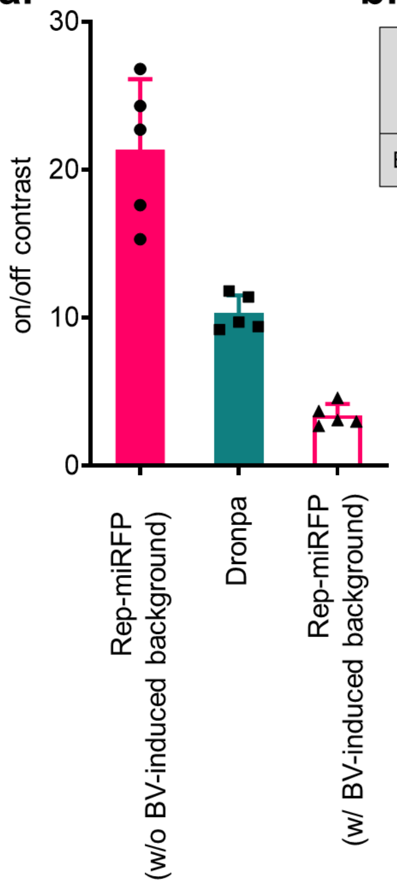

b.

Measurement Condition	Rep-miRFP (w/o BV-induced background)		Rep-miRFP (w/ BV-induced background)	
	Off state	On state	Off State	On state
BV in medium?	-	+	+	+

Supplementary Figure 7. On/off contrast of Rep-miRFP in the absence or presence of BV-induced fluorescent background. **a.** Comparison of on/off contrast with Dronpa. n = 5 cells. **b.** Experiment conditions for measuring on/off contrast of ensemble fluorescence in fixed HeLa cells. For calculating on/off contrast in the absence of BV-induced background, off-state fluorescence was measured before adding BV in the medium. Then, on-state fluorescence was measured after adding BV. For calculating on/off contrast in the presence of BV-induced background, off-state fluorescence was measured with BV already added in the medium, but Rep-miRFP were switched off by 640 nm irradiation. Then, on-state fluorescence was measured after recovery in the dark via chromophore renewal.

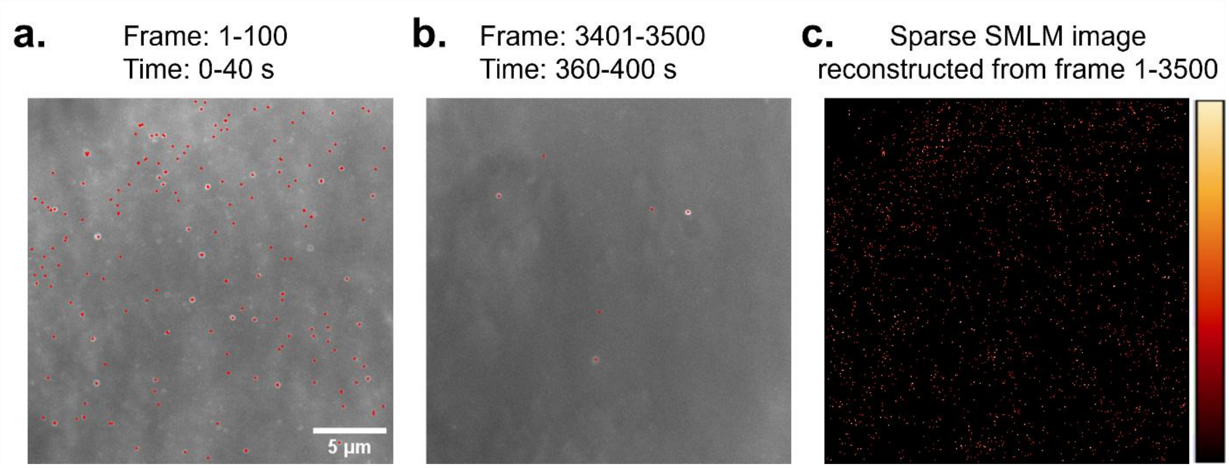

Supplementary Figure 8. Live-cell SMLM imaging with Rep-miRFP after long-term incubation with high concentration of BV. Live COS-7 cells expressing Rep-miRFP-Sec61β was incubated with 30 μM BV for 24 hours before the experiment. Image recording began after single emitters appeared under continuous 640 nm laser irradiation. **a.** Single emitters (highlighted by red dots) localized between frame 1-100. **b.** Single emitters (highlighted by red dots) localized between frame 3401-3500. Localizable molecules were mostly exhausted at this time point. **c.** Sparse SMLM image reconstructed from the entire track of frame 1-3500.

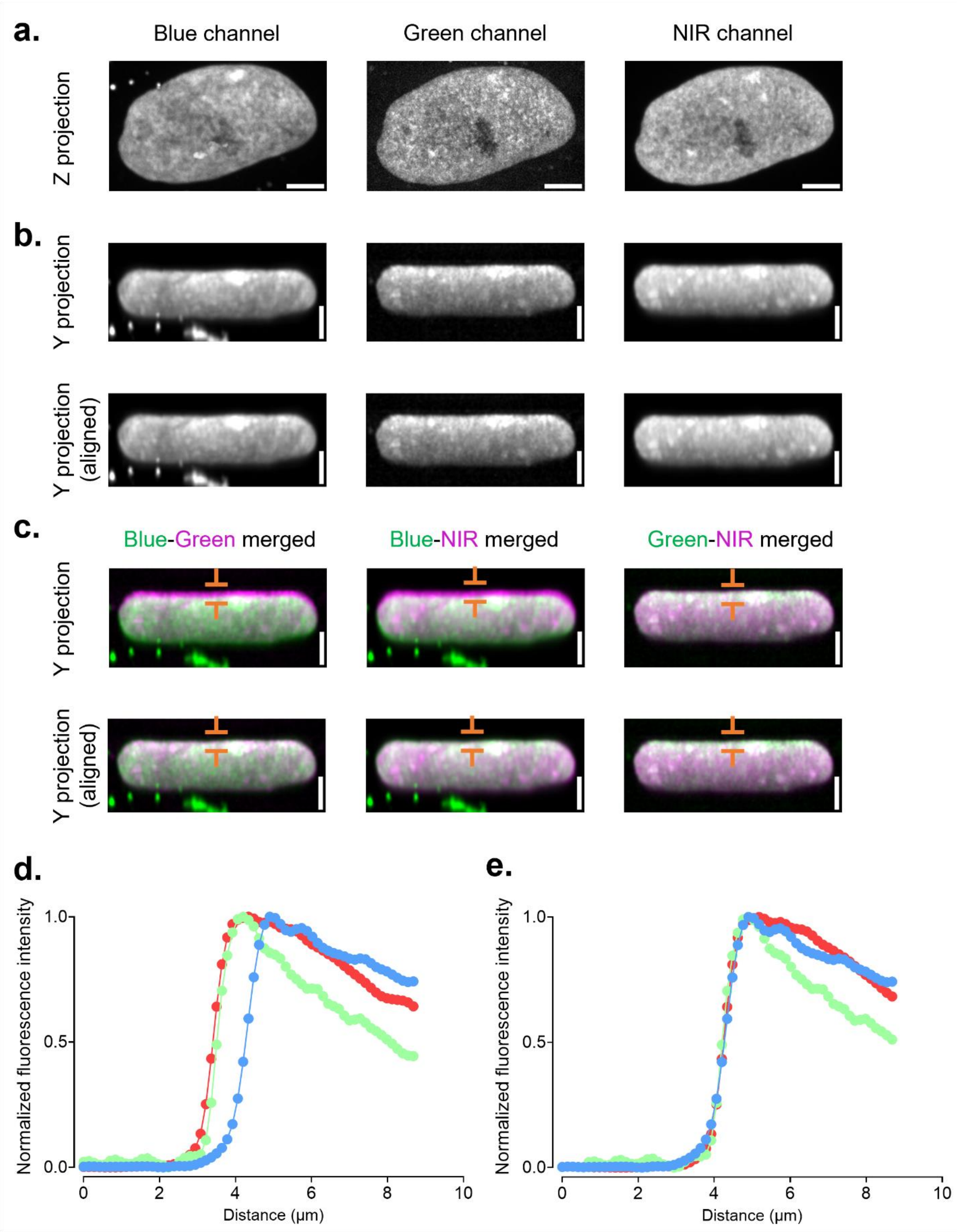

Supplementary Figure 9. Axial chromatic aberration measurement and correction. The cell nucleus was used as a marker for channel alignment and chromatic aberration estimation of the

Apo TIRF 100×/1.49 oil immersion objective lens. The nucleus was labeled by Hoechst staining in blue channel, SPOON-H2B in green channel, miRFP720-H2B in NIR channel. Co-transfected HeLa cells were fixed before image acquisition. **a.** Z-stack confocal image projections of the nucleus acquired with a step size of 0.14 μm. **b.** Y-axis projections of each channel before and after alignment. **c.** Merged images before and after alignment. **d.** Intensity line profile before alignment, measured on top edge of the nucleus (orange arrows in panel c). **e.** Intensity line profile after alignment, measured on top edge of the nucleus (orange arrows in panel c). The estimated Z-axis offsets when aligned to the NIR channel were +0.14 μm for green channel and +0.84 μm for blue channel, which were compensated in multiplexed SMLM.
