# Supplementary material for: Near-infrared PAINT localization microscopy via chromophore replenishment of phytochrome-derived fluorescent tag
Source: Commun Biol. 2024 Apr 18;7:473. doi: 10.1038/s42003-024-06169-7 (PMC11026395; doi:10.1038/s42003-024-06169-7)
Supplement: Supplementary file 2 — Description of Additional Supplementary Files [file 42003_2024_6169_MOESM2_ESM.pdf]

Description of Additional Supplementary Files

File name: Supplementary Data
Description: The source data for the main graphs.

File name: Supplementary Movie 1
Description: Transition of Rep-miRFP from ensemble fluorescence to single emitters under continuous 640 nm laser irradiation in the presence of 0.5 μM BV. Fluorescence intensity was normalized between the highest and lowest intensity within each frame to aid visualization.

File name: Supplementary Movie 2
Description: Induction of non-specific intracellular fluorescence by BV in live HeLa cells upon plasma membrane permeabilization by digitonin.

File name: Supplementary Movie 3
Description: Raw and background-subtracted single-emitter image tracks of Rep-miRFP.
